# Mechanism of hypoxia-induced damage to the mechanical property in human erythrocytes—band 3 phosphorylation and sulfhydryl oxidation of membrane proteins

**DOI:** 10.3389/fphys.2024.1399154

**Published:** 2024-04-19

**Authors:** Qinqin Yang, Dong Chen, Chungong Li, Runjing Liu, Xiang Wang

**Affiliations:** Key Laboratory of Biorheological Science and Technology, Ministry of Education, College of Bioengineering, Chongqing University, Chongqing, China

**Keywords:** resveratrol, band 3 crosslinking, hypoxia, phosphorylation, erythrocyte deformability

## Abstract

**Introduction:** The integrity of the erythrocyte membrane cytoskeletal network controls the morphology, specific surface area, material exchange, and state of erythrocytes in the blood circulation. The antioxidant properties of resveratrol have been reported, but studies on the effect of resveratrol on the hypoxia-induced mechanical properties of erythrocytes are rare.

**Methods:** In this study, the effects of different concentrations of resveratrol on the protection of red blood cell mor-phology and changes in intracellular redox levels were examined to select an appropriate concentration for further study. The Young’s modulus and surface roughness of the red blood cells and blood viscosity were measured via atomic force microsco-py and a blood rheometer, respectively. Flow cytometry, free hemoglobin levels, and membrane lipid peroxidation levels were used to characterize cell membrane damage in the presence and absence of resveratrol after hypoxia. The effects of oxida-tive stress on the erythrocyte membrane proteins band 3 and spectrin were further investigated by immunofluorescent label-ing and Western blotting.

**Results and discussion:** Resveratrol changed the surface roughness and Young’s modulus of the erythrocyte mem-brane, reduced the rate of eryptosis in erythrocytes after hypoxia, and stabilized the intracellular redox level. Further data showed that resveratrol protected the erythrocyte membrane proteins band 3 and spectrin. Moreover, resistance to band 3 pro-tein tyrosine phosphorylation and sulfhydryl oxidation can protect the stability of the erythrocyte membrane skeleton net-work, thereby protecting erythrocyte deformability under hypoxia. The results of the present study may provide new insights into the roles of resveratrol in the prevention of hypoxia and as an antioxidant.

## 1 Introduction

Mature red blood cells (erythrocytes) are deformable and transport oxygen and nutrients by forming in a single row through capillaries that are smaller than their diameter, which is essential for the normal physiological activities of mammals. Erythrocytes have no nucleus or inner membrane system, and the lipid bilayer of the red blood cell membrane, embedded proteins, transmembrane proteins, and membrane skeleton network located below the lipid bilayer play decisive roles in regulating the deformability, oxygen-carrying, and oxygen-releasing abilities of red blood cells (Mohandas and Gallagher, 2008; Himbert and Rheinstädter, 2022). The deformability of erythrocytes depends on both shear resistance and tensile resistance. Furthermore, their deformity is also related to the integrity and stability of the membrane skeleton network. The binding of membrane proteins to the cytoskeleton allows erythrocytes to maintain good membrane surface area and cell integrity (Diez-Silva et al., 2010; Rossi et al., 2019). Band 3 and spectrin are the main proteins in the erythrocyte membrane cytoskeletal network. Band 3 is composed of two domains: a, abbreviated as the “transmembrane domain,” is a binding site for the membrane skeleton, glycolytic enzymes, and deoxyhemoglobin, and b, cytosolic binding domains that primarily form erythrocyte anion-exchange channels and contribute to carbon dioxide transport. (phosphofructokinase, aldolase, and glyceraldehyde-3-phosphate dehydrogenase) (Lux, 2016; Pretini et al., 2019) ensures the average elasticity of erythrocyte membranes and the deformability of red blood cells. Previous studies have shown that abnormal proteins on the erythrocyte membrane can lead to the appearance of spherical cells, changes in cell osmotic pressure, and impairments in the ability of cells to perform normal circulation functions (Vercellati et al., 2022). Abnormal membrane proteins not only affect the physiological and biochemical properties of cells but also lead to blockage of blood circulation. Studies have shown that red blood cell deformability is associated with many clinical manifestations (Shin et al., 2007; Caimi et al., 2018; Jasenovec et al., 2019). Moreover, the deformability of erythrocytes has been increasingly studied for blood storage and transfusion *in vitro* (Koch et al., 2019; Yoshida et al., 2019; Barshtein et al., 2020; 2021; Karafin et al., 2023), in addition to focusing on blood type, transfusion volume and blood quality. Based on the good deformability of red blood cells, the detection methods mainly include AFM to detect cell elasticity and EI index (Huisjes et al., 2018; Carelli-Alinovi et al., 2019; Yang et al., 2019; Lopes et al., 2023; Spinelli et al., 2023).

This amazing deformability of red blood cells has undergone various tests, the most important of which is the attack of reactive oxygen. When too much oxidant is produced, or when the endogenous antioxidant defense is inefficient, this balance may be disturbed and cells are attacked (Remigante and Morabito, 2022). Oxidative stress promotes human erythrocyte senescence (Remigante et al., 2022) Reactive oxygen species have been reported to cause protein kinase C (PKC) activation(Shan et al., 2020), PKC regulates erythrocyte deformability by phosphorylating bands 4.1, 4.9, and adducin, changing the binding affinity of these proteins to other membrane skeleton proteins (Govekar and Zingde, 2001). And, when exposed to high altitude hypoxia, hemorrhagic shock, or other hypoxic injuries, the deformability of erythrocytes is impaired. Further, the process by which cells respond *in vivo* to the hypoxic environment through regulation is multidimensional and involves complex signal transduction processes (Taylor and Moncada, 2010); for example, erythrocyte transglutaminase 2 (eTG2) has been found to act as an erythrocyte protein stabilizer that regulates oxygen delivery to alleviate hypoxia (Xu et al., 2022). Furthermore, researchers have suggested that a hypoxic environment is beneficial for the storage of red blood cells (Rabcuka et al., 2022; Kriebardis, 2023). Although inflammatory signals *in vivo* have an adaptive mechanism in response to hypoxia, inflammation levels beyond those that can be self-regulated lead to an inflammatory response that produces many free radicals and reactive oxygen species (ROS) (Pham-Huy et al., 2008; Pandey and Rizvi, 2010a). Due to the lack of biosynthetic mechanisms, erythrocytes cannot respond to hypoxic injury through protein synthesis, and thus, erythrocyte damage will accumulate uncontrollably (Badior and Casey, 2021). In addition, it has been found that oxidative stress can drain from red blood cells and trigger the damage of adjacent cells and tissues, leading to vascular injury (Carelli-Alinovi et al., 2015). Clinically, the interaction between hypoxia and inflammation may lead to inflammatory-mediated metabolic and cardiovascular comorbidities (Maxwell et al., 1999; Walmsley et al., 2014). Many of the free radicals generated by hypoxia destroy the redox system *in vivo*, triggering membrane lipid peroxidation, and destroying the network structure of the erythrocyte membrane, which directly reduces the deformation ability of erythrocytes (Tasali and Ip, 2008; Quercioli et al., 2010; Cavailles et al., 2013; Walmsley and Whyte, 2014; Pham et al., 2021). Hypoxia-related damage to erythrocytes is irreversible, so identifying additional extracts or drugs to antagonize hypoxia is necessary and meaningful.

Resveratrol (3,5,4’-trihydroxy-trans-stilbene, Res) is a polyphenol stilbene present in red wine, grapes, berries, and peanuts and also a plant antitoxin produced by plants to resist external stimuli, which is highly desirable (Renaud and De Lorgeril, 1992; Weiskirchen and Weiskirchen, 2016; Gorabi et al., 2021). As a nonflavonoid bioactive polyphenolic compound, resveratrol has great potential in disease prevention and treatment. First, resveratrol is a powerful antioxidant, and it has been shown that resveratrol has better antioxidant effects in individuals that are young and middle aged (Galiniak et al., 2019; Malaguarnera, 2019; Santos et al., 2023). In addition, many studies have confirmed that resveratrol has anti-inflammatory, antiaging, and antitumor effects; protects blood vessel walls and the heart; and inhibits low-density lipoprotein oxidation (Saiko et al., 2008; Fei et al., 2018; Li et al., 2019a; 2019b; Huang et al., 2020; Chen and Musa, 2021). Resveratrol can even improve obesity and the condition of being overweight (Durazzo et al., 2019; Tabrizi et al., 2020). These effects of resveratrol are exerted in blood circulation and are related to its concentration in the blood. Resveratrol relies on circulation in the blood to reach its target site. In recent years, several studies have focused on the effects of resveratrol on red blood cells (Suwalsky et al., 2015; Gallardo et al., 2019; Bardyn et al., 2021; Meleleo, 2021), and these effects have been studied from different perspectives. We have focused mainly on the effects and potential underlying mechanisms of resveratrol on the mechanical properties and membrane proteins of circulating red blood cells. Salidroside (SAL), the main medicinal component of salidroside rosea, has long been used as a medicinal material and is a promising antioxidant preparation. If resveratrol has the same effects as salidroside, additional options for clinical treatment could be provided.

The aim of this study was to investigate the mechanism of action of resveratrol on the cytoskeleton network of erythrocytes damaged by hypoxia. Based on the findings of previous studies, further elucidating the potential protective effects of resveratrol against hypoxia-induced damage to erythrocytes is necessary.

## 2 Materials and methods

This study was conducted in accordance with the Declaration of Helsinki, and the protocol was approved by the Ethics Committee of Chongqing University Cancer Hospital. All adult volunteers provided informed consent for inclusion before they participated in the study (protocol code CZLS2023261-A).

### 2.1 Blood samples

Venous blood samples, taken from the forearm vein of healthy adult volunteers, were treated with the anticoagulant sodium citrate (15 IU/mL). The collected blood was centrifuged at 800 × g for 3 min to separate the plasma and red blood cells. Then, the red blood cells were washed with phosphate-buffered saline (PBS), the supernatant was completely removed with a pipette, and the pellet was centrifuged 3 times for 2 min each time. Finally, 0.45 L/L hematocrit was retained, to which citrate phosphate dextrose solution was added. This solution can provide nutrients to the red blood cells and maintain the cellular osmotic pressure (main ingredients: citric acid, sodium citrate, glucose, and phosphate). The resulting mixture was a suspension of red blood cells.

### 2.2 Resveratrol concentration selection

#### 2.2.1 Red blood cell morphology

Suspension of red blood cells were divided into three groups for this part of the experiment: a control group (normoxic conditions, 37°C, 120 min), a hypoxic group {in which a red blood cell suspension was placed in an anoxic chamber [95% N_2_, 5% CO_2_ (Boyd et al., 1980; Dillon and Waldrop, 1992; Bruhn et al., 2018), 37°C, 120 min]}, and a different concentration treatment group. In the treatment group, the red blood cell suspension was mixed with resveratrol so that the final concentrations of resveratrol were 10 μM, 40 μM, 70 μM, 100 μM, and 150 μM. Then, the erythrocytes were incubated with the resveratrol at 37°C for 60 min in normoxic conditions, followed by hypoxia treatment for 120 min.

Afterward, a 1 mL sample was taken from each group for centrifugation at 800 × g for 3 min, followed by washing with PBS (800 × g) 3 times for 2 min each time. Then, after the cells were diluted in PBS to a concentration of 10^4^ cells/mL, 10 μL was placed on a slide and imaged using an Olympus IX71 microscope equipped with a 63/1.25 oil immersion objective and a CCD camera (Olympus, Tokyo, Japan).

Images of 50 erythrocytes from each experimental group were visually inspected under an electron microscope (4-5 photographs) to determine the proportions of discocytes and echinocytes.

#### 2.2.2 Redox levels

To further evaluate the concentration of resveratrol that can protect red blood cells, we performed assays to determine the level of oxidoreductase and extent of membrane lipid peroxidation in red blood cells from each group using kits. The activity of catalase (CAT) was determined by measuring the absorbance at 405 nm by the ammonium molybdate method (Góth, 1991), activity of CAT was determined as follows: CAT (U/mL)=((OD_control_–OD_treatment_) *271*60)/0.1, 271 is constant, 60 is reaction time (s), and 0.1 is sample volume.

Superoxide dismutase (SOD) activity was determined by measuring the absorbance at 450 nm by the 2-(4-iodophenyl)-3-(4-nitrophenyl)-5-(2,4-disulfonic acid phenyl)-2H-tetrazolium salt, disodium salt (WST-1) method. Lipid peroxidation of the erythrocyte membrane was evaluated by measuring the content of malondialdehyde (MDA), the product of lipid peroxidation, via the thiobarbituric acid (TBA) method (Liu et al., 2022). TBA reactive material levels were estimated by measuring the absorbance at 532 nm. Activity of MDA was determined as follows: MDA (nmol/mL) = (OD_treatment_-OD_control_)/(OD_standard_-OD_blank_)*10 nmol/mL/0.15 mL, 10 nmol/mL is concentration of standard, 0.15 mL is sample volume.

The methemoglobin (MetHb) content was used to assess the degree of cellular oxidation. Two samples from each experimental group. One sample was added to the hemoglobin test solution for measurement of the absorbance at 540 nm, and the other sample was added to the methemoglobin test solution to measure the absorbance at 630 nm and 602 nm. Activity of MetHb was determined as follows: MetHb =[(OD630 nm-0.14*OD602 nm)/OD602 nm*1.67] * OD540 nm*367.7, where 0.14,1.67 and 367.7 are constants determined by the kit company.

The calculations for all assays were performed according to manufacturer’s instructions of the kits provided by the Nanjing Jiancheng Institute of Biological Engineering. (http:Njjcbio.com/product.asp).

### 2.3 Effect of resveratrol on the rheological properties of erythrocytes

In a subsequent experiment, we established six experimental groups: the healthy red blood cells group (control), the hypoxia group (hypoxia), the healthy red blood cells add resveratrol group (C-Res), the healthy red blood cells add salidroside group (C-SAL), the hypoxic red blood cells add resveratrol group (H-Res), and the hypoxic red blood cells add salidroside group (H-SAL), with the salidroside group serving as the active control and the hypoxia group serving as the negative control. The working concentration of resveratrol and salidroside was 70 μM (purchased from Beijing Solarbio Science & Technology Co., Ltd.).

Blood is a non-Newtonian fluid, and the viscosity of blood at high shear rates is related to erythrocyte deformability, whereas at low shear rates, blood viscosity is related to erythrocyte aggregation (Shiga et al., 1990; Beris et al., 2021). Isolated plasma was added to washed red blood cells from each experimental group to simulate whole blood for whole blood viscosity testing (0.45 L/L hematocrit). The viscosity of each sample suspension was measured by an automatic hemorheology rapid detector (FASCO-5010DX, Chongqing Weiduo Technology Co., Ltd., China) to determine the deformability and aggregation of red blood cells. The hemorheometer was heated to and maintained at 37°C, after which the channel was cleaned. Samples from each group were then placed into tubes for whole blood viscosity determination. At the end of the test, the channel was cleaned again.

### 2.4 Influence on erythrocyte deformability

Atomic force microscopy (AFM; BRUKER, Dimension ICON010803, United States) was used to measure the Young’s modulus and surface roughness of the cells. Pretreated erythrocytes were resuspended in PBS and added to polylysine-treated glass slides. Peak force quantitative nanomechanical mapping (PFQNM) mode was selected for AFM calibration. Measurements were started after a single red blood cell was observed under the microscope using a soft-cone silicon nitrided cantilever with an elastic constant of 0.92 N/m (F), a probe angle of 18°, and a probe radius of 23.1 nm. In PFQNM mode, three kinds of images can be obtained: a high sensor, peak force error and DMT modulus. The Young’s modulus of the cells was determined in the DMT Modulus channel using Nanoscale analysis software. In addition, the 5 μm × 5 μm region of the cell was selected in the DMT Modulus channel to determine the surface roughness.

For each experiment, 30 cells from each group were randomly examined under a microscope.

AFM was performed based on the Sneddon model (Sneddon, 1965) formula:

$$F=\frac{\pi }{2}\left(\frac{E}{1-v^{2}}\right)\tan\;\left(\alpha \right)\delta^{2}$$

where E is the elastic or Young’s modulus, *v* is the Poisson ratio (assumed to be 0.5), α is the opening angle and δ is the indentation depth. Here, F = 0.92 N/m.

The surface roughness was calculated with the following formula (https://www.bruker.com/):

$$R_{q}=\sqrt{\frac{\sum Z_{i}^{2}}{N}}$$

where R_q_ is the mean square root roughness, N is the number of sampling points, and Z is the height of the ith sampling point.

### 2.5 Detection of erythrocyte membrane damage—FHb, MetHb, and MDA

For the detection of free hemoglobin (FHb) in the supernatants from different treatment groups, the absorbance at 510 nm was measured after different color development reactions. First, the relationship between the optical density and the concentration of the standard substance was determined. Then, 0.15 mL of supernatant was collected from each group, and 2.5 mL of chromogenic agent was added. In the control group, 0.15 mL of double distilled water and 2.5 mL of chromogenic agent were added. Each mixture was heated in a water bath at 37°C for 20 min, after which the optical density was measured at a wavelength of 510 nm. The free hemoglobin concentration was calculated according to the standard curve. Then FHb concentration was experimentally calculated according to: FHb (mg/L) = 126.03*(OD_treatment_-OD_blank_) + 3.5041, 126.03, and 3.5041 were based on a standard curve.

The method for detecting MetHb and MDA were consistent with that described in Section 2.2.2.

All the parameters and calculation methods used were measured using kits provided by Nanjing Jiancheng Bioengineering Institute (Njjcbio.com/product.asp) according to the manufacturer’s instructions.

### 2.6 Eryptosis detection by flow cytometry

Differently treated erythrocytes were centrifuged at 800 × g for 5 min, after which the supernatant was discarded and the cells were collected and resuspended in PBS. Then, 1–5 × 10^5^ cells were taken and centrifuged again at 800 × g for 5 min, and the supernatant was discarded. Cell survival was detected using an Annexin V-FITC apoptosis detection kit (China, http://www.solar.bio.com). Briefly, 500 μL of binding solution was added to the cellular precipitate and mixed gently. Then, 5 μL of Annexin V-FITC was added, and the mixture was incubated for 10 min at 25°C in the dark. The CytoFLEX assay was performed immediately (Beckman Coulter, United States).

### 2.7 Determination of erythrocyte membrane sulfhydryl and glutathione levels

Both the reduced glutathione (GSH) molecules in the erythrocytes and the sulfhydryl groups in the erythrocyte membrane proteins can react with 5,5'-dithiol-di-nitrobenzoic acid (DTNB), and the resulting compounds have a maximum absorption peak at 412 nm. The contents of GSH and sulfhydryl groups in membrane proteins can be measured via visible spectrophotometry (Aksenov and Markesbery, 2001).

### 2.8 Immunofluorescence and image analyses

Cells were fixed by mixing 4% paraformaldehyde and 0.01% glutaraldehyde at a 1:1 ratio and then washed with PBS at 1200 rpm 3 times for 2 min each time. Then, 0.01% Triton X-100 was used to permeabilize the red blood cell membranes, after which the cells were washed with PBS at 1200 rpm 3 times for 2 min each time. Bovine serum albumin (BSA) was used to block the other sites on the membrane so that subsequent antibodies could bind specifically. After the corresponding antibody was selected, the cells were incubated with mouse monoclonal spectrin antibody and rabbit monoclonal band 3 antibody (Abcam, UK, No. ab2808; Abcam, UK, No. ab108414) diluted 1:200 in 5% BSA for 2 h at room temperature. The cells were washed with PBS three times (two minutes each time). Then, the cells were incubated with secondary antibodies (Alexa Fluor 488 goat anti-mouse, No. A0428 and Alexa Fluor 488 goat anti-rabbit, No. A0423; Beyotime, China), diluted with 5% BSA at a 1:400 dilution, and washed three times with PBS. Finally, the cells were imaged to observe fluorescence using an Olympus IX71 microscope equipped with a 63/1.25 oil immersion objective and a CCD camera (Olympus, Tokyo, Japan).

### 2.9 Western blot analysis

Rinsed erythrocytes were placed in a cold lysis buffer mixture containing 1 mM MgCl_2_, 1 mM ethylenediaminetetraacetic acid sodium, 10 mM Tris-HCl, and 0.1 mM phenylmethylsulfonyl fluoride (pH 8.0) at 4°C for hemolysis. Erythrocyte membrane proteins were collected after treatment with lysis buffer and centrifugation at 12,000 rpm for 10 min, and protein concentrations were quantified by the Bradford method. The proteins were solubilized in SDS‒PAGE loading buffer with or without dithiothreitol (DTT) at a volume ratio of 4:1, and each mixture was incubated at 100°C for 10 min.

Clear bands were obtained by electrophoresis on a preconfigured polyacrylamide separation gel and a concentrated gel. The proteins were subsequently transferred from the gel to a polyvinylidene fluoride membrane and immunostained with primary antibodies (mouse monoclonal to spectrin, No. S3396, Sigma, Germany, diluted 1:500; rabbit monoclonal to band 3, No. ab108414, Abcam, UK, diluted 1:5000; rabbit monoclonal to β-actin, No. 380624, ZENBIO, China, diluted 1:5000; and rabbit monoclonal to band 3 phospho Y359 and phospho Y21, Abcam, UK, diluted 1:20,000 and 1:10,000. No. ab77236 and No. ab125070). The sections were incubated with anti-mouse (No. 701051) or anti-rabbit (No. 511203) secondary antibodies (ZENBIO, China) diluted 1:10,000, and the proteins were detected via chemiluminescence (Thermo Scientific, United States). Quantification of the immunocomplexes was conducted by lengthwise scanning densitometry using a gel analyzer image processing program (Azure Biosystem, United States).

### 2.10 Statistical analysis

The experimental results are expressed as the mean ± standard deviation. One-way ANOVA was used to compare more than three groups.

Origin 9.0 software was used for statistical analysis. Quantity One was used for gray analysis. FlowJo was used for cell eryptosis analysis.

## 3 Results

### 3.1 Selection of resveratrol concentration—erythrocyte morphology

**FIGURE 1:**
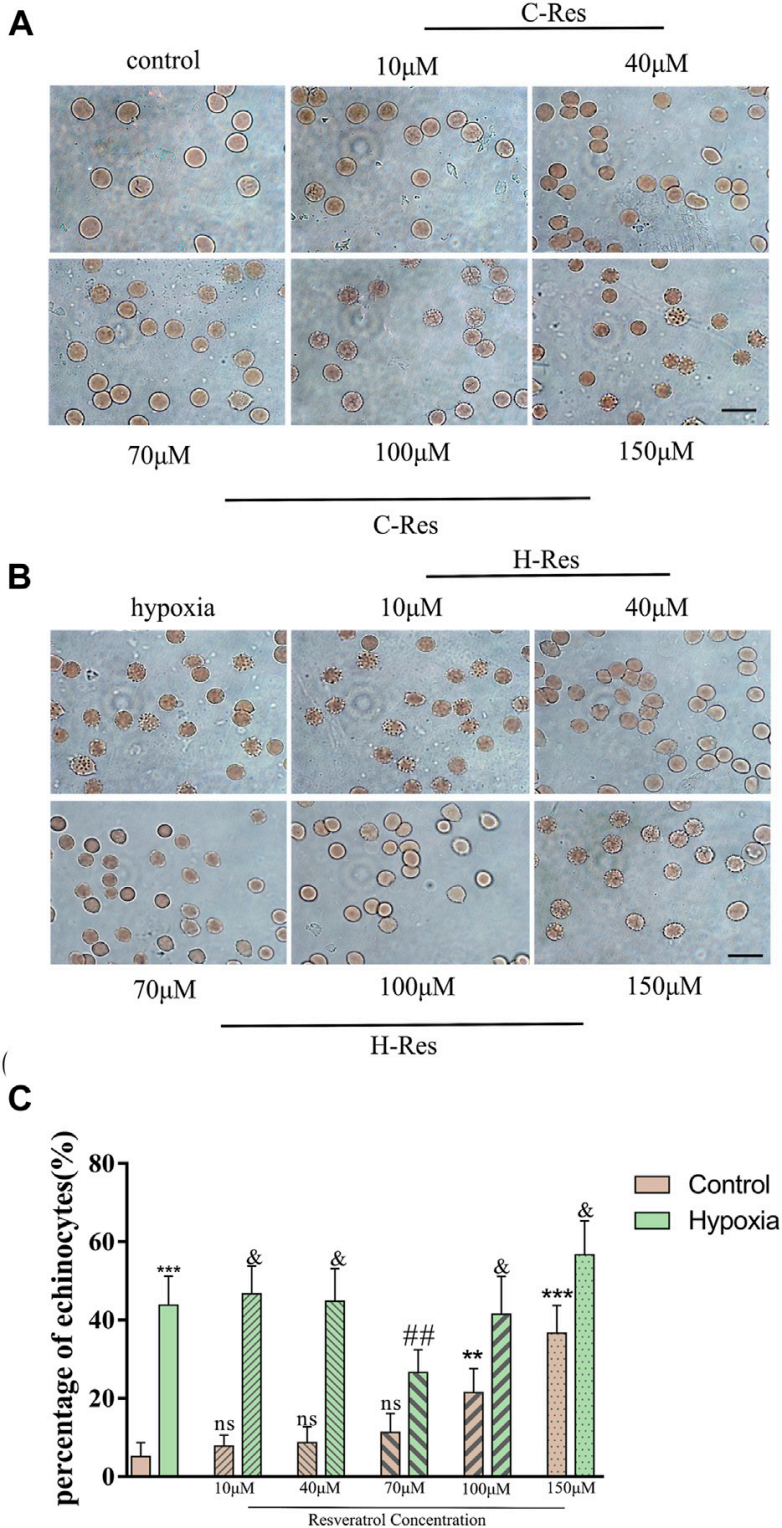
**(A)**, Changes of erythrocyte morphology in different concentrations of resveratrol. **(B)**, Morphological changes of erythrocytes after different concentrations of resveratrol were incubated and treated with hypoxia. Scale bar = 10 μm. **(C)**, Percentage of echinocytes. *n* = 3, compared with the control group, **p < 0.01, ***p < 0.001, ns means not significant; compared with hypoxia group ##*p* < 0.01, & means not significant.

When a red blood cell suspension was placed in a hypoxic environment, echinocytes (spiculated cells) appeared. To determine an appropriate treatment concentration of resveratrol, normal red blood cells were incubated with different concentrations of resveratrol (Figure 1A), and then, some of the cells were subjected to hypoxic conditions (Figure 1B) and observed under a light microscope to determine the resveratrol concentration that had a protective effect on red blood cell morphology.

As shown in Figure 1C, when the resveratrol concentration was increased to 100 μM, the number of echinocytes increased to more than 20%, and at 150 μM resveratrol, 40% were echinocytes. Compared with the normal group, high concentrations of resveratrol (100 μM and 150 μM) were not conducive to protecting cell morphology (*p* < 0.01, *p* < 0.001). Compared with the hypoxia group, 70 μM resveratrol protected cell morphology (*p* < 0.01). Moreover, there were no significant differences between the low (10 μM and 40 μM) and high (100 μM and 150 μM) resveratrol concentration groups and the hypoxia group.

### 3.2 Selection of resveratrol concentration—effect on redox levels in red blood cells

To determine the optimal working concentration of resveratrol, the levels of CAT, SOD, MDA, and MetHb in normal and hypoxic erythrocytes incubated with resveratrol were measured.

**FIGURE 2:**
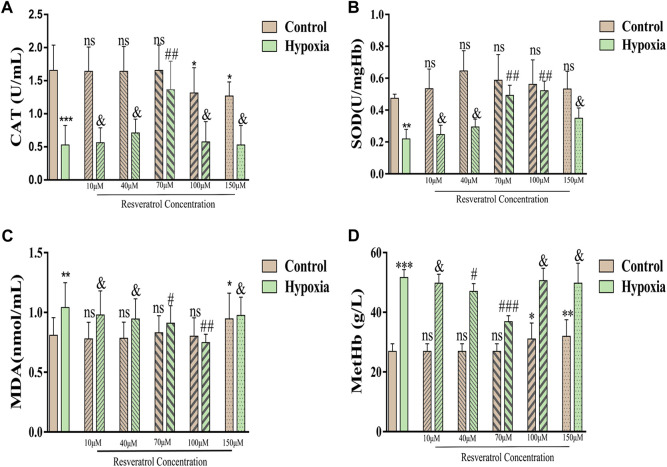
**(A)**, Changes of CAT contents after incubation with different concentrations of resveratrol. Among them, the Y-axis in panel A represents CAT activity, defined as the amount of 1umol hydrogen peroxide catabolized per milliliter of serum per second as a unit of activity; **(B)**, Changes of SOD content, the Y-axis is the SOD activity, which is the corresponding enzyme amount when the SOD inhibition rate reaches 50%; **(C)**, Changes in MDA content; **(D)**, Changes in MetHb content. Values are expressed as the mean ± SD. Compared with control group, **p < 0.01, ***p < 0.001, ns means not significant; Compared with hypoxia group, ##*p* < 0.01, & means not significant. *n* = 3.

Compared with the control group, the levels of CAT (Figure 2A) and SOD (Figure 2B) were significantly lower after hypoxia treatment (*p* < 0.001; *p* < 0.01), but the MDA (Figure 2C) and MetHb (Figure 2D) levels were higher (*p* < 0.01; *p* < 0.001). After incubating normal erythrocytes with different concentrations of resveratrol, the CAT content gradually decreased with increasing concentration (100 μM and 150 μM) and was significantly different from that in the control group (*p* < 0.05); however, the SOD content did not change significantly with increasing resveratrol concentration. With respect to the MDA content, when the concentration of resveratrol was increased to 150 μM, the MDA content increased and was significantly different from that in the control group (*p* < 0.05). When the concentration of resveratrol was 100 μM or 150 μM, the MetHb content was significantly higher than that in the control group (*p* < 0.05; *p* < 0.01).

After hypoxia treatment after incubation with 70 µM resveratrol, the levels of CAT and MetHb were significantly different from those in the hypoxia group (*p* < 0.01; *p* < 0.001). The levels of SOD and MDA after treatment with 70 μM and 100 μM resveratrol were significantly different from those in the hypoxia group (*p* < 0.01; *p* < 0.01; *p* < 0.05; *p* < 0.01).

Considering the changes in red blood cell morphology and CAT, SOD, MDA, and MetHb levels, we selected 70 μM resveratrol as the optimal concentration for use subsequent experiments.

### 3.3 Effect of resveratrol on erythrocyte aggregation and deformation

**FIGURE 3:**
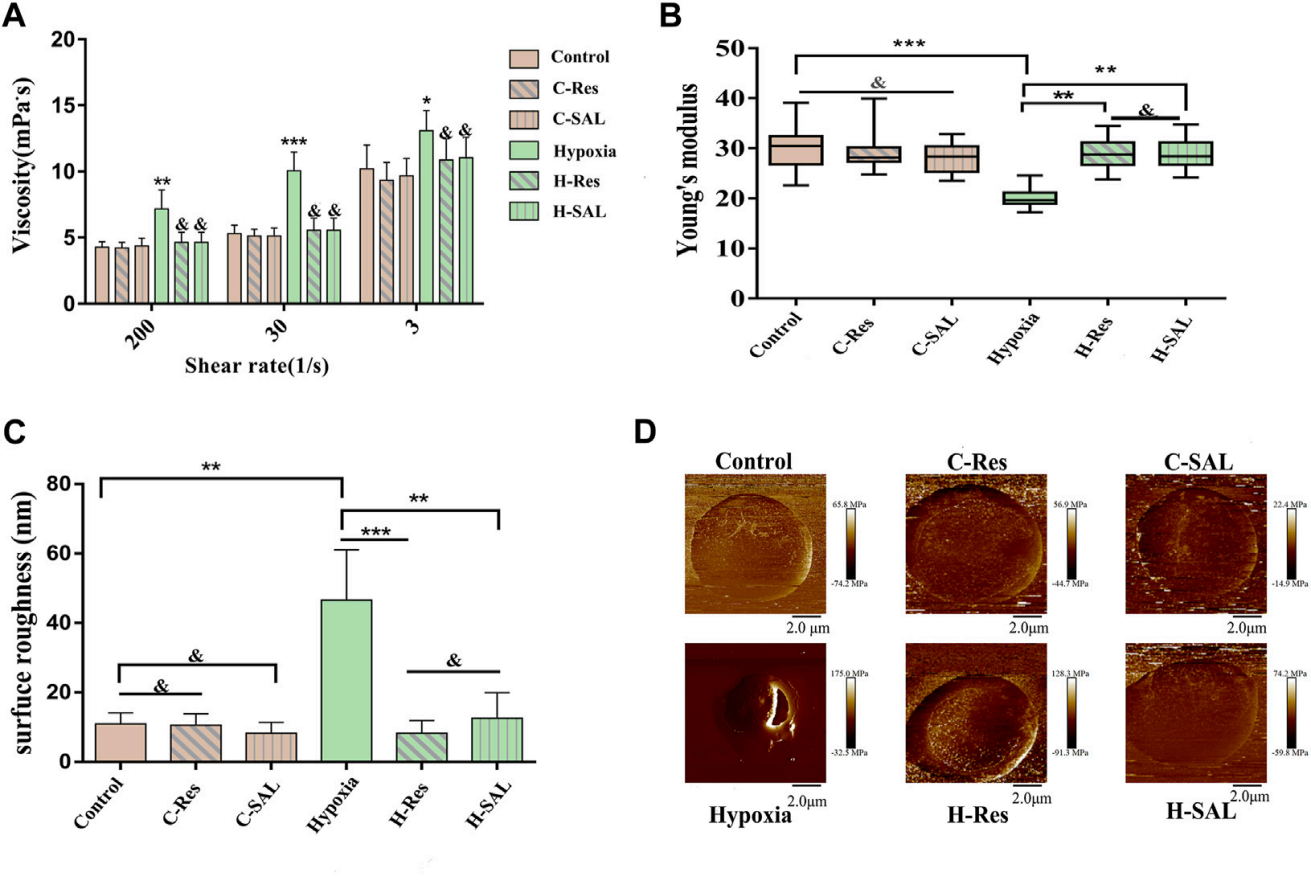
**(A)**, The viscosity of erythrocyte suspension was measured at different shear forces to indirectly characterize the aggregation and deformability of erythrocyte. Compared with control group, *p < 0.05, **p < 0.01, and ***p < 0.001. Compared with the hypoxia group, & means there is no significant difference. **(B)**, Changes in the Young’s modulus and **(C)** surface roughness of erythrocyte reflect the deformability of cells. surface roughness was measured over the 5 μm × 5 μm area. **p < 0.01, ***p < 0.001 and and means no significant difference. *n* = 3. **(D)**, DMT modulus, representative images of individual cells in each group were selected.

Whole-blood viscosity can reflect the aggregation and deformability of red blood cells. According to the results in Figure 3A, we found that the simulated whole-blood viscosity of the hypoxia group was significantly greater than that of the normal group under both high (*p* < 0.05), medium (*p* < 0.001), and low shear rate (*p* < 0.05) conditions. When resveratrol and salidroside were added, the whole-blood viscosity did not significantly change compared with that of the control group, and resveratrol achieved the same effect as salidroside.

To investigate the cellular mechanical properties of hypoxic erythrocytes after resveratrol treatment, 30 single cells from each sample were selected for AFM measurements, which were repeated three times. The whole experiment consisted of six independent replicate tests, and the resulting data collation is shown in Figure 3B. Neither the presence of resveratrol nor the presence of salidroside affected the mechanical properties of red blood cells. After hypoxia treatment, the Young’s modulus of the red blood cells decreased significantly (*p* < 0.001). However, after resveratrol or salidroside were added, the Young’s modulus increased (*p* < 0.01), and there was no obvious difference between them. The surface of the normal red blood cells was smooth. After hypoxia treatment, the surface roughness of the red blood cells increased, and after resveratrol and salidroside treatment, the surfaces of the red blood cells were smoother than those of the hypoxia group, as shown in Figure 3C.

Figure 3D presents the DMT modulus diagram, which shows representative images of single cells selected from each group.

### 3.4 Protection of the erythrocyte membrane by resveratrol after hypoxia

**FIGURE 4:**
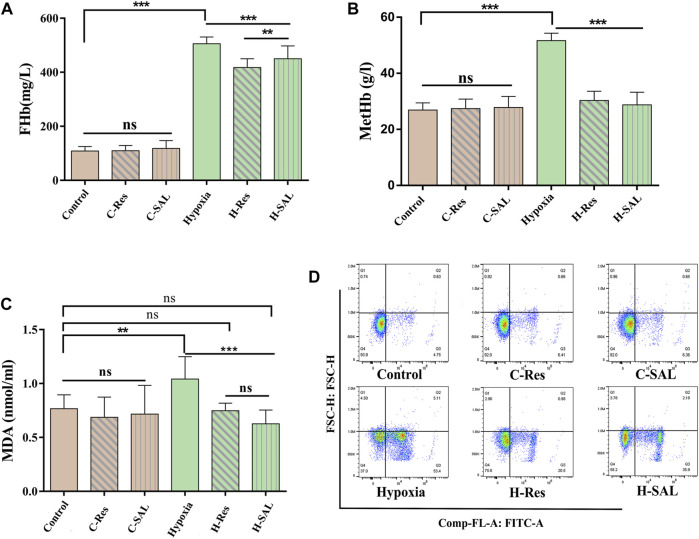
Erythrocyte membrane damage was measured by **(A,B)** free hemoglobin and methemoglobin content in erythrocyte suspensions, and **(C)** membrane lipid peroxidation levels. **(D)** Effect of resveratrol on eryptosis induced by hypoxic stimulation, *n* = 3. Values are expressed as the mean ± SD. **p < 0.01, ***p < 0.001 and ns means no significant difference.

The levels of FHb, MetHb, and MDA were measured to investigate the effect of resveratrol on hypoxic injury in erythrocytes. As shown in Figure 4, the contents of FHb (Figure 4A), MetHb (Figure 4B) and MDA (Figure 4C) did not change significantly in control red blood cells after the addition of resveratrol or salidroside. After hypoxia, the FHb, MetHb and MDA levels increased (*p* < 0.01; *p* < 0.001; *p* < 0.001). In the presence of resveratrol and salidroside, the FHb, MetHb, and MDA contents were lower than those in hypoxia group.

Flow cytometry was used to analyze whether resveratrol could protect red blood cells from eryptosis. The results (Figure 4D) show that when normal red blood cells were incubated with resveratrol and salidroside, there was no significant change in the number of eryptotic cells. After hypoxia, the percentage of cells binding Annexin V increased from 6.12% to 63.01%. However, under hypoxic conditions, resveratrol and salidroside significantly reduced the percentage of cells binding Annexin V from 63.01% to 24.44% and 41.86%, respectively.

### 3.5 Effect of erythrocyte membrane proteins

**FIGURE 5:**
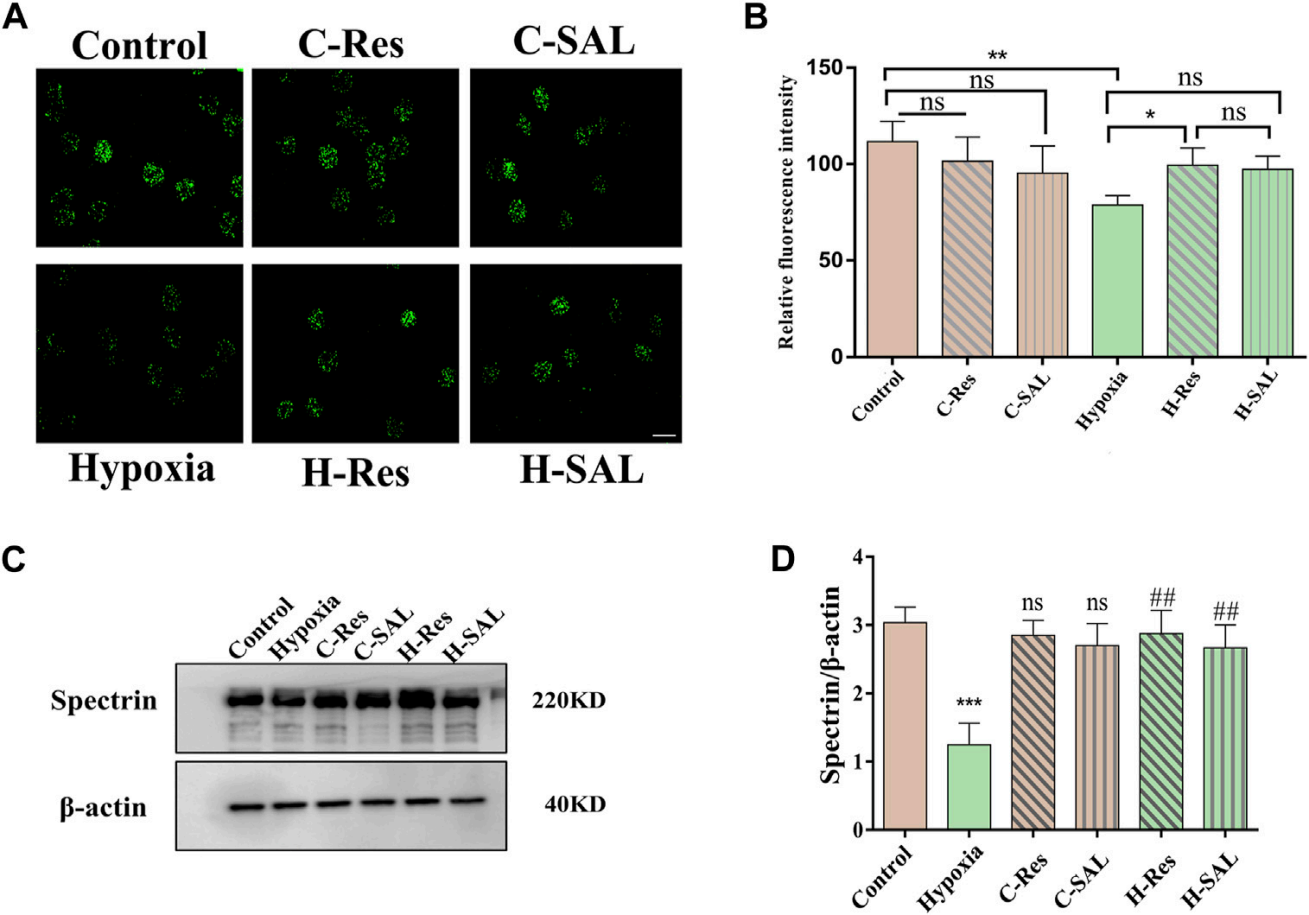
**(A)** Immunofluorescence staining of spectrin protein in different groups was performed and **(B)** the fluorescence intensity was analyzed, scale bar = 10 μm. **(C)** Western blotting was used to analyze the effect of resveratrol on spectrin protein after hypoxic stress in the absence of DTT. **(D)** Spectrin western blot density was quantified, and β-actin was used as an internal reference. *n* = 3. Compared with the control group, ***p < 0.001, ns means no significant difference. Compared with the hypoxia group, ##*p* < 0.01.

Spectrin is the main skeletal component of the erythrocyte membrane that plays an important role in maintaining erythrocyte deformability. Immunofluorescence localization analysis (Figure 5A) showed that resveratrol and salidroside had no effect on the expression of the protein spectrin, although hypoxia decreased the average fluorescence intensity of the spectrin protein (Figure 5B; *p* < 0.01). After incubation with resveratrol, the fluorescence intensity of the cells was significantly greater than that in the hypoxia group (*p* < 0.05).

Western blotting confirmed the effects of hypoxia and resveratrol supplementation on the spectrin protein level (Figure 5C). Hypoxia led to a decrease in the spectrin protein content (Figure 5D; *p* < 0.001), but after the addition of resveratrol and salidroside to the hypoxia treatment group, the protein content of spectrin was significantly greater than that in the hypoxia group (Figure 5D; *p* < 0.01; *p* < 0.01), indicating that resveratrol could resist the damage to spectrin caused by hypoxic conditions.

### 3.6 Effect of resveratrol on erythrocyte membrane -SH levels

**FIGURE 6:**
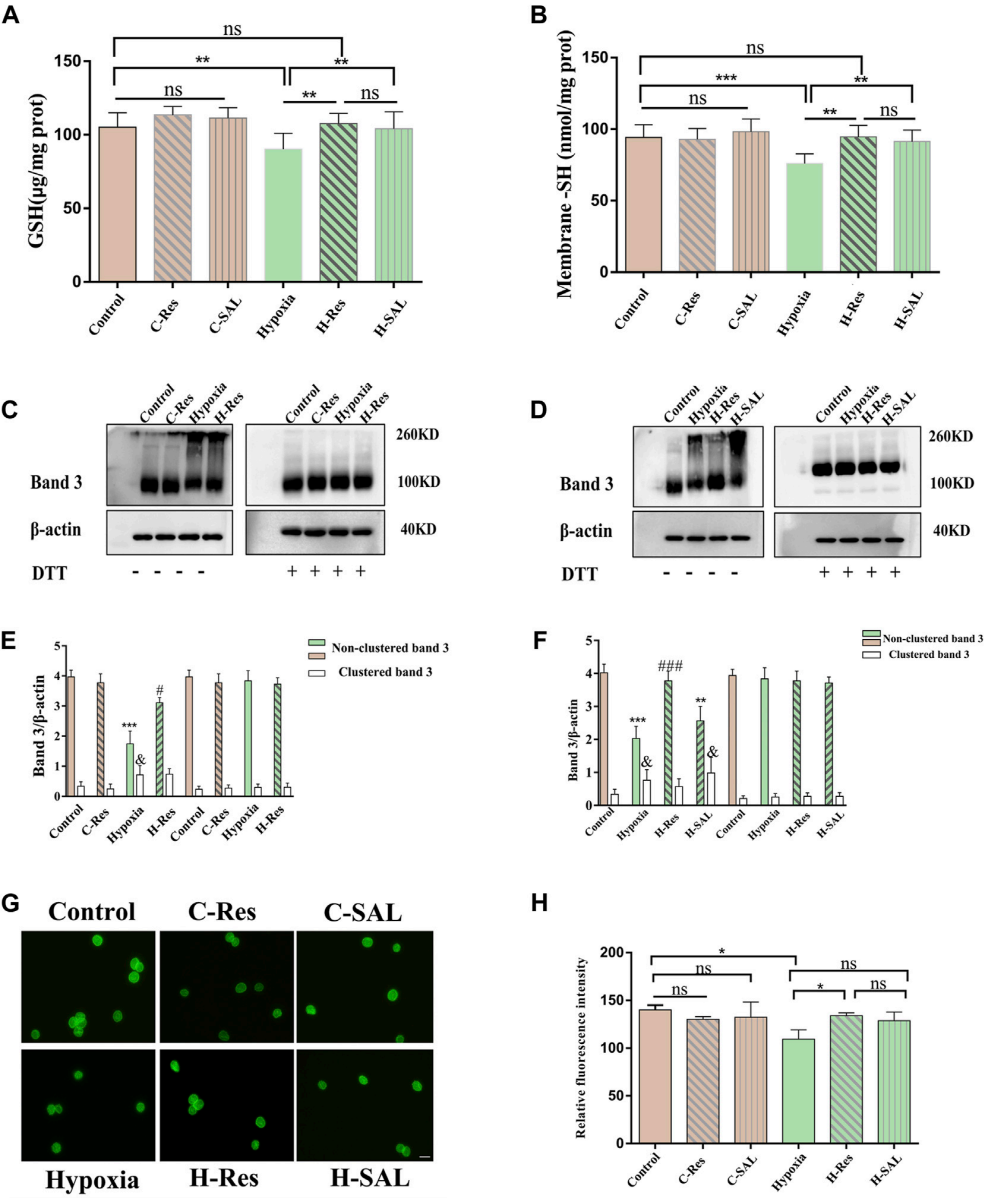
Protection of thiol groups by resveratrol **(A)** erythrocyte glutathione and **(B)** changes in protein thiol content on membranes. Values are expressed as the mean ± SD. **p < 0.01, ***p < 0.001 and ns means no significant difference. **(C,D)** The effect of resveratrol on the crosslinking sum of hypoxia stress-related band 3 was analyzed by western blotting in the presence or absence of DTT, **(E,F)** The band 3 cluster immunoblot density was quantified, and β-actin was used as an internal reference. Values are expressed as the mean ± SD. Compared with the unclustered control group, ***p < 0.001; Compared with the unclustered hypoxia group, ###*p* < 0.001; Compared with the clustered control group, & *p* < 0.05. **(G)** Cellular immunofluorescence staining for band 3 protein. Scale bar = 10 μm. **(H)** The average fluorescence intensity of immunofluorescence staining of band 3 protein in different groups was analyzed, *p < 0.05, and ns means no significant difference. *n* = 3.

Compared with those in the control group, the intracellular GSH and membrane-SH contents in the hypoxia group were significantly lower (Figure 6A, *p* < 0.01; Figure 6B, *p* < 0.001), but the GSH and -SH contents were significantly greater in the presence of resveratrol (*p* < 0.01; *p* < 0.01) and salidroside (*p* < 0.01; *p* < 0.01). After hypoxia treatment, there was no significant difference in the GSH or -SH content between the groups incubated with resveratrol and salidroside.

Resveratrol may regulate the deformability of hypoxic erythrocytes under hypoxic conditions by regulating the cross linking of band 3. To explore this possibility, we examined the status of band 3 after incubation under hypoxic conditions or with resveratrol in the presence or absence of DTT. Figure 6C–F shows that the band 3 protein content in the hypoxia group decreased significantly in the absence of DTT (*p* < 0.05, *p* < 0.001). Additionally, this protein was partially oxidized to a high polymer (*p* < 0.05, *p* < 0.05) but reduced back to a band between 90 and 100 kDa in erythrocytes after incubation with DTT. After incubation with resveratrol in the absence of DTT, the band 3 protein content was significantly greater than that in the hypoxia group (*p* < 0.05; *p* < 0.001) the polymers were reduced or absent with no significant difference compared with the crosslinked band 3 in the control group (Figures 6C–F). Hypoxia-associated oxidative stress leads to damage caused by the free sulfhydryl groups in membrane proteins, thereby inducing the oxidation of band 3 proteins through the formation of reducible intermolecular and/or intramolecular disulfide bonds.

Immunofluorescence localization analysis of the membrane proteins was also performed (Figures 6G, H). Compared with those in the control group, the relative fluorescence intensity of the band 3 protein in normal erythrocytes incubated with resveratrol and salidroside did not significantly change. After hypoxia, the relative fluorescence intensity of the band 3 protein on the erythrocyte membrane was significantly lower than that in the control group (*p* < 0.05; *p* < 0.01). After the addition of resveratrol, the relative fluorescence intensity of the band 3 protein was significantly greater than that in the hypoxia group (*p* < 0.05; *p* < 0.05). After the addition of salidroside, the relative fluorescence intensities of band 3 and the contractile protein were not significantly different from those in the hypoxia group. There was no significant difference between the resveratrol group and the salidroside group after hypoxia treatment.

### 3.7 Analysis of band 3 protein tyrosine phosphorylation

**FIGURE 7:**
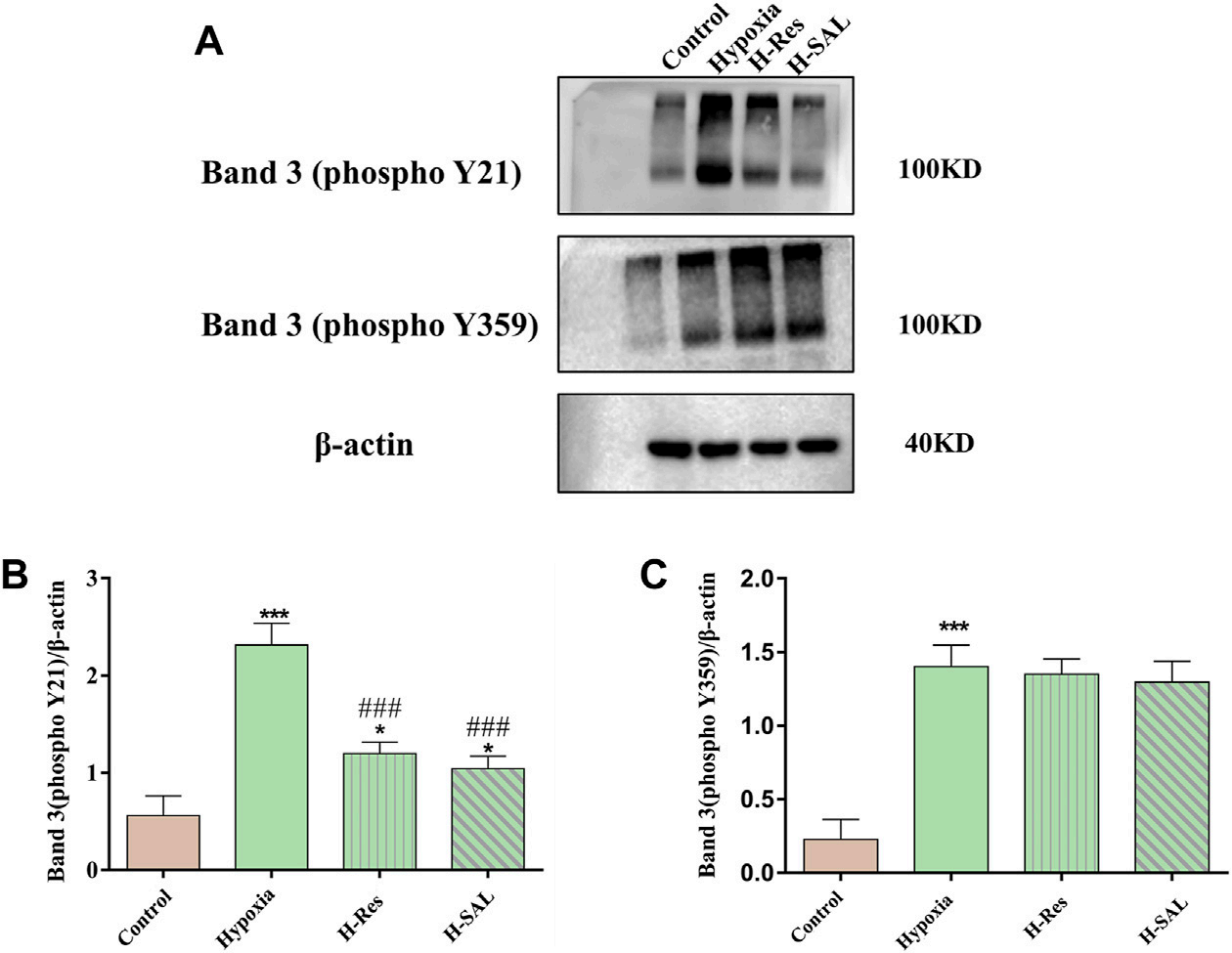
**(A)** Western blot was used to detect the phosphorylation of tyrosine 21 and 359 in band 3, **(B,C)**, and the western blot band was quantified using actin as an internal reference. Compared with the control group, *p < 0.05, ***p < 0.001. Compared with the hypoxia group, ###*p* < 0.001. *n* = 3.

Western blot analysis of tyrosine phosphorylation at positions 359 and 21 in the band 3 protein (Figure 7A) showed that band 3 phosphorylation was induced after erythrocytes were subjected to hypoxia (Figures 7B, C; *p* < 0.001; *p* < 0.001). After resveratrol or salidroside were added, compared with that in the hypoxia group, the phosphorylation of band 3 at position 21 was decreased (Figure 7B; *p* < 0.001, *p* < 0.001). However, after the addition of resveratrol or salidroside, there was no significant difference in phosphorylation at position 359 of the band 3 protein compared with that in the hypoxia group (Figure 7C).

## 4 Discussion

Resveratrol protect the morphological integrity of red blood cells and prevent damage to the red blood cell membrane, the oxidation of membrane sulfhydryl groups, damage to the protein spectrin, and the phosphorylation of the protein band 3 under hypoxia. All of these are important events involved in the stabilization of the mechanical properties of the membrane.

The first step of this study was to test different concentrations of resveratrol (from 10 μM to 150 μM) to rule out the possible effect of high dose of resveratrol on red blood cells. In this experiment, the optimal working concentration of resveratrol was evaluated from four aspects: red blood cell morphology, red blood cell REDOX level (CAT and SOD), red blood cell membrane lipid peroxidation level (MDA) and MetHb. As shown in Figure 1A, increased concentrations of resveratrol above 100 μM resulted in the appearance of acanthocytes; Conversely, it was shown in 1b that resveratrol at 40–70 μM had the best protective effect on red blood cell morphology caused by hypoxia. Similarly, by examining the response of REDOX level in normal erythrocytes to different concentrations of resveratrol in Figure 2, we found that the content of CAT decreased at 100 μM, while the content of CAT in hypoxic erythrocytes showed a trend of first increasing and then decreasing, with the highest content of CAT at 70 μM. In addition, there was no significant change in SOD content in normal erythrocytes with the increase of resveratrol concentration, but the SOD content in hypoxic erythrocytes increased with the increase of resveratrol concentration, and the SOD content was the highest at 70 μM and 100 μM. In the experiments with MDA and MetHb, the same pattern emerged. This suggests that excessive antioxidants may exhibit pro-oxidative (Giordano et al., 2020) and pro-membrane lipid peroxidation effects. Based on these findings, we chose 70 μM resveratrol to investigate the possible mechanism in the hypoxia model, which is also close to the concentration selected by other investigators in different disease models (Horn et al., 2007; Saiko et al., 2008; Komorowska et al., 2023; Ei et al., 2024; Fu et al., 2024).

The results of atomic force microscopy showed that resveratrol had a statistically significant effect on the increase of Young’s modulus of red blood cells induced by hypoxia in Figure 3. In addition, the blood viscosity increased at a high shear rate after hypoxia as determined by hemorheology, which clinically indicates that the deformability of red blood cells was poor. Moreover, viscosity also increased at a low shear rate, suggesting that the red blood cells were aggregated. These results all suggest that resveratrol is involved in hypoxia-induced changes in erythrocyte deformability. We further found that resveratrol could decrease the expression of free hemoglobin and methemoglobin in the cytoplasm of hypoxic erythrocytes, protect the integrity of erythrocyte membrane, and reduce the expression of MDA in hypoxic erythrocytes. This also proves that resveratrol protects erythrocyte membrane integrity and inhibits membrane lipid peroxidation. The structure, composition, and fluidity of the erythrocyte membrane affect erythrocyte deformability. Erythrocytes are among the most vulnerable cells to oxidative stress. Unsaturated fatty acids on the cell membrane undergo oxidative attacks, leading to membrane lipid peroxidation and decreased membrane fluidity (Mohandas and Chasis, 1993; Ranjekar et al., 2003). Human erythrocytes are rich in sulfhydryl groups, and the importance of the -SH groups on the erythrocyte membrane in the overall cellular redox balance has been emphasized. Hypoxia results in increased sulfhydryl oxidation, which is inhibited by resveratrol, indicating that resveratrol exerts a very rapid protective effect on the oxidation of membrane sulfhydryl groups (Pandey and Rizvi, 2010b). In previous studies, resveratrol was shown to stimulate the production of heme oxygenase-1 (HO-1), whose degradation promotes the oxidation of oxidized heme to produce bilirubin, thereby eliminating oxygen free radicals (Doré, 2002; Zhuang et al., 2003; Juan et al., 2005) and protecting the body from the adverse effects of various environmental toxins (Latruffe et al., 2015; Vestergaard and Ingmer, 2019). Resveratrol can effectively reduce the expression of inflammatory factors such as interleukin-6 (IL-6), which are activated by hypoxia, and activate the production of the deacetylase Sirt1 (Gomes et al., 2018).

Changes in erythrocyte membrane function during hypoxia are associated with changes in membrane biophysical properties (George et al., 2010; 2013). Spectrin is a major component of the cytoskeleton, and the spectrin-based membrane skeleton essentially maintains the integrity and mechanical properties of the cell membrane and the cytoskeletal network that supports cell shape. In our experiments, hypoxia-induced spectrin degradation led to destabilization of the membrane skeleton network, altered discocyte morphology, and decreased cell deformability. In addition, our study showed that crosslinked band 3 forms oligomer protein complexes under hypoxic conditions. This high-molecular-weight protein is involved in hypoxia-associated oxidative stress, leading to damage by free sulfhydryl groups in membrane proteins during hypoxia, thereby inducing the oxidation of the band 3 protein through the formation of reversible intermolecular and/or intramolecular disulfide bonds. No high-molecular-weight oligomeric proteins were found after resveratrol treatment, suggesting that resveratrol inhibits the oxidation of free sulfhydryl groups. The band 3 protein cluster was shown to be a recognition site generated during erythrocyte decay (Arese et al., 2005), which induces recognition and clearance from the circulation by macrophages. Sulfhydryl groups on the erythrocyte membrane are closely related to erythrocyte viscoelasticity and osmotic fragility (Pandey and Rizvi, 2010b). Combined with the deformability analysis, the protection of hypoxia-induced protein aggregation by resveratrol may be one of the mechanisms by which resveratrol improves red blood cell deformability.

The function of the band 3 protein on the erythrocyte membrane is regulated by protein tyrosine phosphatases (PTP), spleen tyrosine kinase (syk) and Src family tyrosine kinase (Lyn), Y-phosphorylation/dephosphorylation is likely involved in the regulation of several erythrocyte functions (Minetti et al., 1998, p. 3; Brunati et al., 2000) Tyrosine phosphorylation of band 3 is induced by many stimuli, such as aging, malaria, and oxidative stress (Ferru et al., 2011, p. 3; Spinelli et al., 2023). In the present study, hypoxia induced the formation of disulfide bonds from the free sulfhydryl groups on the erythrocyte membrane. The formation of disulfide bonds attenuated the dephosphorylation of PTP, and in this way, a series of acidic amino acids in syk and lyn surrounded the band 3 protein and removed most of it from the membrane skeleton. Previous studies have shown that phosphorylation of band 3 on the erythrocyte membrane reduces binding to anchor proteins (Pantaleo et al., 2009; Ferru et al., 2011, p. 3), increasing the mobility of band 3 and its phosphorylation and promoting the dissociation of band 3 from anchor proteins and its aggregation, which seriously damages the stability of the skeleton network and reduces the deformability of erythrocytes. Thus, band 3 tyrosine phosphorylation promotes band 3 crosslinking, leading to reduced erythrocyte aggregation and deformability. Due to the dissociation of ankyrin from band 3 by the peroxidation of membrane lipids and tyrosine phosphorylation of band 3, the immobile band 3 becomes mobile and is prone to cluster formation. Resveratrol may act as a regulatory fragment to alter the affinity between the anchoring protein and band 3 by changing the phosphorylation state of band 3 in a hypoxic environment.

*In vitro* and *in vivo* phosphorylation of band 3 in human erythrocytes is catalysed by syk and Src-associated tyrosine kinases, lyn acts on tyrosines located in the thiol (SH)-binding domain of the band 3 protein, and tyr21 and tyr359 are the main binding sites for syk and lyn, respectively (Yannoukakos et al., 1991; Brunati et al., 2000). Bands were analyzed by immunoblotting, under hypoxic conditions, the sulfhydryl group of the band 3 protein is oxidized, leading to phosphorylation of tyr21 and tyr359. Phosphorylation of tyr21 causes the band 3 protein to undergo a change in conformation, which further promotes tyr359 phosphorylation. Resveratrol attenuates the degree of phosphorylation of the band 3 protein by protecting its molecular structural stability and membrane sulfhydryl groups from oxidative attack, especially by protecting tyr21. Reduced phosphorylation associated with syk and lyn by resveratrol is consistent with previous findings (Kotha et al., 2006; Chung et al., 2019; Lewis et al., 2024).

## Data Availability

The original contributions presented in the study are included in the article/Supplementary Material, further inquiries can be directed to the corresponding author.
